# Burned-out with burnout? Insights from historical analysis

**DOI:** 10.3389/fpsyg.2022.993208

**Published:** 2022-09-08

**Authors:** Renzo Bianchi, Katarzyna Wac, James Francis Sowden, Irvin Sam Schonfeld

**Affiliations:** ^1^Norwegian University of Science and Technology (NTNU), Trondheim, Norway; ^2^University of Geneva, Geneva, Switzerland; ^3^Flinders University, Adelaide, SA, Australia; ^4^City College of New York (CUNY), New York City, NY, United States

**Keywords:** burnout, occupational depression, methodology, diagnosis, construct proliferation, historical analysis

## Abstract

Fierce debates surround the conceptualization and measurement of job-related distress in occupational health science. The use of burnout as an index of job-related distress, though commonplace, has increasingly been called into question. In this paper, we first highlight foundational problems that undermine the burnout construct and its legacy measure, the Maslach Burnout Inventory (MBI). Next, we report on advances in research on job-related distress that depart from the use of the burnout construct. Tracing the genesis of the burnout construct, we observe that (a) burnout’s definition was preestablished rather than derived from a rigorous research process and (b) the MBI has little in the way of a theoretical or empirical foundation. Historical analysis suggests that the burnout construct was cobbled together from unchallenged personal impressions and anecdotal evidence before getting reified by the MBI. This state of affairs may account for many of the disconcerting problems encountered in burnout research. We close our paper by presenting the Occupational Depression Inventory (ODI), a recently developed instrument reflective of a renewed approach to job-related distress. The ODI has demonstrated robust psychometric and structural properties across countries, sexes, age groups, occupations, and languages. The instrument addresses job-related distress both dimensionally and categorically. A dimensional approach can be useful, for instance, in examining the dynamics of etiological processes and symptom development. A categorical approach can serve screening and diagnostic purposes and help clinicians and public health professionals in their decision-making. It is concluded that the ODI offers occupational health specialists a promising way forward.

## Introduction

Job-related distress constitutes a major public health concern (Hassard et al., 2018; Howard et al., 2021). The phenomenon harms individuals, organizations, and society as a whole, with yearly costs in dozens of billions of US$/€ in Western countries (European Agency for Safety and Health at Work et al., 2015; Hassard et al., 2018). Over the last decades, *burnout* has become a commonly employed index of job-related distress (Schaufeli, 2017). Burnout has been defined as a stress-induced syndrome reflecting symptoms of exhaustion, cynicism, and inefficacy (Maslach et al., 2001). The Maslach Burnout Inventory (MBI) embodies this three-component definition (Maslach and Jackson, 1981; Maslach et al., 1996, 2016). The MBI has been the most widely used measure of burnout and has played a decisive role in shaping burnout research (Maslach et al., 2001; Schaufeli, 2017; Schonfeld et al., 2019b).

While burnout has gained considerable popularity since the introduction of the MBI in the early 1980s (Maslach and Jackson, 1981), occupational health specialists have identified worrying shortcomings in the construct (Rotenstein et al., 2018; Schwenk and Gold, 2018; Bianchi et al., 2021a; Meier, 2022; Saul and Nikolitch, 2022). Despite their gravity, these shortcomings have largely been overlooked, and few efforts have been devoted to investigating their root causes. In this paper, we highlight foundational problems that undermine the burnout construct and report on recent advances in research on job-related distress. These recent advances may help the researcher, practitioner, and policymaker communities address job-related distress more effectively.

## Foundational problems affecting the burnout construct

The importance of the MBI for the definition and legitimization of the burnout construct has often been underlined (Maslach et al., 2001; Friberg, 2009; Schaufeli et al., 2009). For instance, Schaufeli (2017) noted: “Initially, the scientific community deemed burnout a ‘pseudoscientific’ or ‘fad’ concept and denounced it as ‘pop psychology’, but this soon changed after the introduction of the MBI” (p. 108). The MBI has been used in a vast majority of the studies involving burnout (Schaufeli and Enzmann, 1998; Schaufeli et al., 2009; Schonfeld et al., 2019b). The instrument has been so influential as to inspire the ICD-11’s description of burnout among the “factors influencing health status or contact with health services” (World Health Organization, 2022).^1^ However, historical analysis reveals that the foundations of the MBI are not nearly as solid as suggested by the instrument’s hagiographers. The studies that led to the development of the MBI were rudimentary in their designs and analyses (e.g., uncontrolled observations having indeterminable reliability and validity), lacked methodological safeguards (e.g., to reduce the impact of investigators’ expectations and preconceived beliefs), and showed little anchorage in the literature on stress-related conditions available at the time (Friberg, 2009; Bianchi and Schonfeld, 2018; Bianchi and Sowden, 2022). The MBI was neither firmly grounded in clinical research nor based on sound theorizing (Schaufeli, 2003). As put by Schaufeli and Enzmann (1998):

“…burnout is what the MBI measures. This tautology is a serious problem since…the MBI has been developed inductively by factor-analy[z]ing a rather arbitrary set of items. What would have happened if other items had been included? Most likely, other dimensions would have appeared!” (p. 188)

Observations of Schaufeli and Enzmann (1998) have broad ramifications. They suggest that *the three-component definition of burnout may be an artifact of the MBI’s problematic development process*. Because the definition of burnout attached to the MBI has been the point of reference for the entire domain of burnout research, including the alternative conceptualizations and operationalizations of the entity, this state of affairs is of concern. Schaufeli and Enzmann’s (1998) observations resonate with the difficult question, raised by Friberg (2009), of whether the *burnout syndrome* was “invented” rather than “discovered” (p. 553).

Interestingly, the developers of the MBI themselves cast doubt on burnout’s definition when recommending that exhaustion, cynicism, and inefficacy be analyzed and interpreted separately due to “limited knowledge about the[ir] relationships” (Maslach et al., 1996, p. 5). Such a recommendation undercuts the idea that burnout is a *syndrome—*i.e., a combination of co-occurring symptoms forming a unified entity (American Psychiatric Association, 2013). Crucially, the recommendation formulated by the MBI developers creates a contradiction between burnout’s conceptualization and operationalization and leaves MBI users with a double-bind dilemma. To respect burnout’s syndromal definition, investigators have to contravene the operational prescription of examining exhaustion, cynicism, and inefficacy separately; to respect the operational prescription of examining exhaustion, cynicism, and inefficacy separately, investigators have to contravene burnout’s syndromal definition. The recommendation made by the MBI developers has serious implications. If exhaustion, cynicism, and inefficacy are three separate entities to be treated individually, then the burnout construct loses its raison d’être (Bianchi and Schonfeld, 2021b; Bianchi and Sowden, 2022). Burnout is supposed to emerge from the combination of exhaustion, cynicism, and inefficacy symptoms. If the operational prescription is adhered to, then burnout is nowhere to be found. As a corollary, the MBI ceases to be a measure of burnout.

The conditions under which the MBI was developed may account for the surprising symptom scope of the measure (Schaufeli and Enzmann, 1998; Hallsten, 2005). On the one hand, the MBI disregards crucial symptoms of job-related distress, such as cognitive impairment and suicidal ideation; on the other hand, the instrument emphasizes symptoms such as “cynical attitudes,” which are less relevant to stress-induced health alterations and have ambiguous implications for job performance (Taris, 2006; Orton et al., 2012; Bianchi et al., 2019). The content of the MBI has been called into question by many investigators, including Schaufeli and De Witte (2017), who concluded that the MBI lacks “internal coherence” (p. 59). Why exhaustion, cynicism, and inefficacy were considered the most relevant symptoms to characterize stressed-out workers has never been clarified. It should be underlined that Maslach’s three-component definition of burnout elicited dissent early on. As an illustration, Pines, who was a collaborator of Maslach during the pre-MBI phase of burnout research (e.g., Maslach and Pines, 1977; Pines and Maslach, 1980), distanced herself from the characterization of burnout that the MBI crystallized (Pines et al., 1981; Pines and Aronson, 1988).

The longstanding inability of burnout researchers to establish (differential) diagnostic criteria for their entity of interest can be viewed as another sign that the burnout construct fails to capture a coherent and distinct phenomenon (Brisson and Bianchi, 2017; Heinemann and Heinemann, 2017; Oquendo et al., 2019; Bianchi et al., 2021a). The impossibility of diagnosing burnout has been a hindrance to case identification, prevalence estimation, treatment development, workers’ access to compensation (e.g., sick pay), and public health decision-making (Rotenstein et al., 2018; Schwenk and Gold, 2018; Bianchi et al., 2021a).^2^ Fascinatingly, burnout is commonly presented as dramatically prevalent despite the absence of a diagnosis. Without a diagnosis, cases of burnout cannot be identified. Because they cannot be identified, they cannot be counted. Because they cannot be counted, no prevalence estimate can be produced.

## Burnout or depression

Although the nature of burnout has been debated over the years, by today, a large body of evidence has accumulated to indicate that what the pioneers of burnout research regarded as a new and unique condition is best understood as a *depressive* condition (Ahola et al., 2014; Wurm et al., 2016; Schonfeld et al., 2019a,b; Bianchi et al., 2021b; Rotenstein et al., 2021; Verkuilen et al., 2021). This observation is consistent with the well-established finding that depression constitutes a basic response to intractable stress—either work-related or not—in individuals with no history of depression and no noticeable genetic susceptibility to depression (Dohrenwend, 2000; Willner et al., 2013). From a neural standpoint, the stress system is critically involved in the regulation of mood, motivation, cognition, and action (Thase, 2009; Sapolsky, 2021). When adversity is chronically experienced as out of the individual’s control and no rewarding, stressor-neutralizing action is available to the individual, the sustained activation of the stress response plays a crucial role in the emergence of depressed mood, apathy, depressive cognition, and behavioral inhibition (Pryce et al., 2011; McEwen, 2012; Willner et al., 2013; Kunz, 2014; Grahek et al., 2019). In keeping with these findings, occupational health practitioners (including psychiatrists) have long argued that the burnout-depression distinction is nosologically superfluous and therapeutically unworkable (Durand-Moreau and Dewitte, 2015). The longstanding difficulties in characterizing burnout have often been attributed to the presumed “complexity” of the phenomenon. Research on the overlap of burnout with depression suggests that the burnout phenomenon may not be so much complex as ill-conceived.

**Figure 1:**
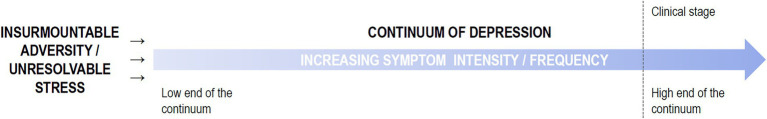
Dimensional view of depression. The clinical stage of depression, at which a depressive disorder can potentially be diagnosed, represents only a fraction of the continuum of depression—its high end.

The burnout-depression distinction has often been promoted based on a faulty conception of depression. The conception in question reduces depression to its clinical stage (at which a depressive *disorder* can be diagnosed), thereby failing to consider that research at the forefront of the field of psychopathology regards depression dimensionally, that is, as a continuum (Pickles and Angold, 2003; Haslam et al., 2012; Bianchi et al., 2021b). The view that burnout may constitute a pre-depressive condition, for instance, overlooks the dimensional aspect of depression and the fact that depressive symptoms vary in degree, from virtually absent symptoms to extremely severe symptoms (see Figure 1).

Attempts at distinguishing burnout from depression have also relied on the view that burnout is a *social* phenomenon whereas depression is an *individual* one (e.g., Pines and Aronson, 1988; Maslach et al., 2001; Epstein and Privitera, 2017). This view has proved to be epistemologically shaky and empirically groundless (Bianchi et al., 2017a; Bianchi and Schonfeld, 2018), in addition to imposing stigma on depressed people. Both burnout and depression can be, and have been, studied from a social and an individual standpoint. Moreover, there is evidence that burnout is, in fact, highly dependent on individual dispositions (Swider and Zimmerman, 2010; Bianchi et al., 2018; Bianchi and Janin, 2019; Rotenstein et al., 2021; Michel et al., 2022). Recent studies relying on relative weight analysis^3^ even found burnout to be more strongly accounted for by personality trait neuroticism than by occupational factors (e.g., Bianchi, 2018; Bianchi et al., 2021b). These findings do not support Maslach and Leiter’s (2016) narrative that “job variables and the organizational context are the prime predictors of burnout” (p. 355).

The overlap of burnout with depression has been further obscured by a misunderstanding of the nature of exhaustion, “the central quality of burnout” (Maslach et al., 2001, p. 402), as an outcome of unresolvable stress. In the context of stress research, exhaustion does not reflect a healthy state of fatigue that would merely result from a temporarily-unrecovered expense of energy. Feeling momentarily exhausted after a lot of effort (e.g., an intense workday) is a normal response. The form of exhaustion that results from unresolvable stress is of a different kind. It emerges from the experience of helplessness and entrapment that unresolvable stress involves (Seligman, 1975; Selye, 1976; Laborit, 1977; Pryce et al., 2011; Kunz, 2014). Individuals get exhausted by prolonged confrontation with stress factors that cannot be controlled and coped with. It is the experience of helplessness and entrapment in the face of adverse conditions that leads to exhaustion. Unsurprisingly, exhaustion is a diagnostic criterion for depression and a common presenting complaint among depressed patients (American Psychiatric Association, 2013).

## More archaeology

An examination of the very first papers of Maslach—the leading developer of the MBI—and her colleagues sheds light on what might be a root cause of the imbroglio surrounding burnout for nearly five decades. Such an examination indeed suggests that burnout’s definition was largely *preestablished*.

In what is known as her first paper on the topic, Maslach (1976) already offered a detailed description and turnkey explanation of burnout *although no systematic research had been conducted on the entity at the time*. No information was provided on the validity and reliability of the modus operandi that was followed to produce the description and explanation in question. In the same article, the author discussed variations in “burnout rates” despite the absence of established criteria for identifying “burned-out” individuals. One year later, exhaustion, cynicism, and inefficacy symptoms were presented as nodal characteristics of burnout without any clear evidence in support of this particular characterization (Maslach and Pines, 1977).

Importantly, no comparative investigations contrasting burnout with already-described stress-related conditions (such as depressive syndromes) can be found in these early publications (Maslach, 1976; Maslach and Pines, 1977) or in the other articles that Maslach and her colleagues published before the release of the MBI (e.g., Maslach, 1978; Pines and Maslach, 1980). It thus appears that the originality of burnout was postulated rather than demonstrated. Moreover, there is no sign that burnout was characterized with the help of stress researchers specializing in behavioral psychology, medicine, or neurobiology. A transdisciplinary dialogue may have put the so-called “discovery of burnout” into perspective and averted the emergence of illusions of novelty regarding the observed phenomenon. The link between (psychosocial) stress and depression, for instance, was already documented in the 1970s (Kollar, 1961; Lundquist, 1961; Forrest et al., 1965; Seligman, 1975; Laborit, 1977; Brown and Harris, 1978; Dohrenwend, 1979).

Historical analysis indicates that burnout’s definition did *not* proceed from a rigorous research process. In the light of burnout’s genesis, the publication of the MBI in 1981 can be seen as the culmination of an exercise of self-confirmation. As a standardized quantitative measure, the MBI gave burnout the patina of scientificity, allowing the construct to establish itself in the academic arena. The low quality of the research on which the MBI rested, as well as the unclear operations and arbitrary decisions that accompanied the instrument’s creation (see Schaufeli and Enzmann, 1998), went largely unnoticed. All in all, the burnout construct and the MBI may owe much of their success to collective amnesia regarding the conditions of their creation.

## Addressing job-related distress differently

In response to burnout’s incapacity to serve as a dependable indicator of job-related distress, new ways to approach job-related distress have begun to emerge. The Occupational Depression Inventory (ODI) was recently developed as part of this renewal (Bianchi and Schonfeld, 2020; Schonfeld and Bianchi, 2021; Bianchi et al., 2022b).^4^

The ODI is designed to assess *work-attributed* depressive symptoms. Unlike the MBI, the instrument exhibits robust psychometric and structural properties (e.g., high factorial validity, strong reliability, measurement invariance across countries, sexes, age groups, occupations, and languages), allows for both continuum-based and diagnostic approaches to job-related distress, and is available free of charge (Bianchi and Schonfeld, 2020; Hill et al., 2021; Bianchi et al., 2022a,b).^5^ The ODI assesses each of the nine core symptoms of major depression (including cognitive impairment and suicidal ideation) *in connection to work* (e.g., “My experience at work made me feel like a failure”). Symptoms are assessed within a two-week time window. The measure can be employed based on its total score, reflecting the severity of work-attributed depressive symptoms, or with the help of an algorithm providing provisional diagnoses of occupational depression. The algorithm references diagnostic criteria for major depression found in the DSM-5 (American Psychiatric Association, 2013). The ODI includes a supplementary turnover-intention item to help evaluate the implications of the reported symptoms (Bianchi and Schonfeld, 2020).

The ODI has demonstrated criterion validity in relation to a variety of variables, including work-life characteristics (e.g., interpersonal conflict at work, job incivility, unreasonable work tasks, unnecessary work tasks, work overload, social support at work, job autonomy, skill development, job recognition, job meaningfulness), general health status, and objective cognitive performance (Bianchi and Schonfeld, 2021a, 2022; Hill et al., 2021; Bianchi et al., 2022a; Schonfeld and Bianchi, 2022). An ODI-based study involving a deep-learning framework recently found occupational depression to be (a) negatively linked to companies’ stock growth and (b) positively linked to states’ economic deprivation (Sen et al., 2022). By assessing symptoms such as depressed mood, fatigue/loss of energy, and feelings of worthlessness, the ODI captures the substance of what the burnout experience purportedly entails. As previously mentioned, the ODI assesses many additional symptoms (e.g., cognitive impairment and suicidal ideation).

Researchers, practitioners, and policymakers need robust indicators to address job-related distress effectively. Robust indicators are central to both knowledge-building and action-taking. They are also important to avoid wasting resources in research settings increasingly marked by sophisticated logistics and reliance on advanced technologies, notably in neurobiological and computational sciences (Wac, 2018). Anchored in the well-established area of stress and depression research, the ODI offers a way to surmount the myriad problems affecting the burnout construct and its measures—most emblematically, the MBI (Table 1).

**Table 1:** Advantages of the Occupational Depression Inventory (ODI) over the Maslach Burnout Inventory (MBI).

	ODI	MBI
Incorporates both dimensional and diagnostic approaches to job-related distress	✓	⛔
Allows for prevalence estimation	✓	⛔
Assesses suicidal ideation—a marker of severe job-related distress	✓	⛔
Has strong clinical and theoretical foundations	✓	⛔
Exhibits sound psychometric and structural properties	✓	⛔
Shows consistent conceptualization and measurement	✓	⛔
Is brief and easy to use	✓	⛔
Is available free of charge	✓	⛔

## Conclusion

Seldom examined, the genesis of burnout calls into question the very foundation of the construct. Historical analysis suggests that the burnout construct was cobbled together from unchallenged personal impressions and anecdotal evidence before getting reified by the MBI. Burnout epitomizes the problem of construct proliferation in psychological science (Le et al., 2010; Bianchi et al., 2017b; Hodson, 2021). The tendency to imprudently add constructs to the scientific marketplace requires more attention given its detrimental consequences for research and practice.

Job-related distress can dramatically affect people’s health and, in the most severe cases, result in suicide (Hassard et al., 2018; Howard et al., 2021). The approach to job-related distress reflected in the ODI promises to help occupational health specialists support personnel more effectively.

## Data availability statement

The original contributions presented in the study are included in the article/supplementary material; further inquiries can be directed to the corresponding author.

## Author contributions

All authors listed have made a substantial, direct, and intellectual contribution to the work and approved it for publication.

## Conflict of interest

The authors declare that the research was conducted in the absence of any commercial or financial relationships that could be construed as a potential conflict of interest.

## Publisher’s note

All claims expressed in this article are solely those of the authors and do not necessarily represent those of their affiliated organizations, or those of the publisher, the editors and the reviewers. Any product that may be evaluated in this article, or claim that may be made by its manufacturer, is not guaranteed or endorsed by the publisher.

## Footnotes

^1^ https://icd.who.int/browse11/l-m/en#/http://id.who.int/icd/entity/129180281

^2^It might be assumed that the *exhaustion disorder* diagnostic category recognized in Sweden constitutes a proxy for "clinical burnout." An exhaustion disorder diagnosis, however, does not require any etiological connection to work.

^3^Relative weight analysis allows investigators to rank a set of predictors according to their importance.

^4^ https://doi.org/10.1037/t84940-000

^5^The MBI has consistently exhibited problematic psychometric and structural properties (see Bianchi et al., 2022b).
